# Leishmaniasis in Sri Lanka: spatial distribution and seasonal variations from 2009 to 2016

**DOI:** 10.1186/s13071-018-2647-5

**Published:** 2018-01-25

**Authors:** Lahiru Sandaruwan Galgamuwa, Samath D. Dharmaratne, Devika Iddawela

**Affiliations:** 1 Department of Parasitology, Faculty of Medicine, University of Peradeniya, Peradeniya, Sri Lanka; 2 Department of Community Medicine, Faculty of Medicine, University of Peradeniya, Peradeniya, Sri Lanka; 3 Institute for Health Metrics and Evaluation, Department of Global Health, School of Public Health, University of Washington, Seattle, USA

**Keywords:** Leishmaniasis, Spatial distribution, Seasonal variations

## Abstract

**Background:**

Leishmaniasis is listed as one of the eight neglected tropical diseases by the World Health Organization and the number of cases in endemic areas has seen a sharp rise in the past decade. More alarmingly, reports have shown that leishmaniasis is spreading to non-endemic areas of the world due to co-infection with HIV. In Sri Lanka, leishmaniasis is considered as a notifiable disease from 2008 and has seen a rising trend of incidence since then. This is the first study describing the burden, seasonal variation and spatial distribution of leishmaniasis in Sri Lanka since the disease has been included as a notifiable disease.

**Methods:**

Data on health statistics from 2009 to 2016 were obtained from published databases maintained by the Epidemiology Unit of the Ministry of Health in Sri Lanka. Climatic data for Sri Lanka were obtained from the Department of Meteorology and the populations in administrative districts were obtained from the Department of Census and Statistics, Sri Lanka. Descriptive spatiotemporal analysis, correlation between leishmaniasis incidence and climatic variables were analyzed using SPSS statistical software.

**Results:**

The total number of people reported with leishmaniasis during the study period was 8487. Cutaneous leishmaniasis is the prominent form in Sri Lanka while few visceral and muco-cutaneous cases were reported. Although leishmaniasis patients were identified from all 25 districts in the island, almost 90% of the total caseload was reported from Anuradhapura, Hambantota, Polonnaruwa, Kurunegala and Matara districts. The highest number of patients was reported from the Anuradhapura district and the highest incidence per 100,000 persons was reported from the Hambantota district. The disease has a seasonal trend, a peak of leishmaniasis occur in July to September in the north-central region and in October to December in the southern region. Maximum temperature, humidity and wind speed are significantly associated climatic variables with leishmaniasis in endemic regions.

**Conclusions:**

Leishmaniasis is an emerging public health problem in north-central and southern Sri Lanka. Public awareness programs for the prevention and control of the disease in endemic regions are essential to reduce the incidence of leishmaniasis.

## Background

Leishmaniasis is endemic in 88 countries, of which 66 are in the Old World, 22 in the New World and 72 of them are developing countries. It has been estimated that 350 million people are at risk of acquiring leishmaniasis, with approximately 0.2 to 0.4 million visceral leishmaniasis (VL) and 0.7 to 1.2 million cutaneous leismaniasis (CL) patients reporting in each year [1]. Changes of natural and man-made environments make leishmaniasis an emerging public health concern due to rapid urbanization, sharp increase in migration, deforestation and adaptation of the *Leishmania* parasite to additional vectors and mammalian hosts [2]. Furthermore, *Leishmania* has emerged as an opportunistic pathogen of HIV-infected adults as well as children [1]. The spectrum of clinical manifestations is large, ranging from the self-healing cutaneous lesions to the more serious, potentially fatal visceralizing form, including the metastasizing muco-cutaneous form, and the post kala-azar dermal leishmaniasis [3].

In Sri Lanka, during 1970s and 1980s, the disease was limited to a few cases among overseas employees returning to the country and remained sporadic in nature [4]. The first locally acquired CL infection was identified in 1992 from Southern Sri Lanka [5]. Since then the number of CL cases identified has increased dramatically. In addition, several sporadic locally acquired cases of mucocutaneous and visceral leishmaniasis have been reported in Sri Lanka [6]. *Leishmania donovani* zymodeme MON -37 was identified as the causative species responsible for CL in Sri Lanka [7]. DNA sequencing and microsatellite analyses have shown that these parasites are closely related to *L. donovani* MON-2 causing VL in the Indian subcontinent [8]. The same parasite, *L. donovani* of the zymodeme MON-37, also causes VL in India, Bangladesh and East Africa [9]. This dermotropism shown by *L. donovani* in Sri Lanka and in few other countries confirms the susceptibility of certain individuals to cutaneous form of leishmaniasis [10, 11]. *Phlebotomus argentipes* (*P. argentipes*) is the main vector of *L. donovani* in Sri Lanka [7, 12]. Recent studies have identified all three members of the *Phlebotomu*s *argentipes* species complex (*P.glaucus, P. argentipes* and *P. annandalei*) in northern Sri Lanka [12, 13]. Distribution of the phlebotomine sand flies depends on geographical differences, abundance of vertebrate hosts, habitat availability and environmental factors such as rainfall and temperature [14, 15]. Hot and humid conditions have played an important role in the distribution of *P. argentipes* in Sri Lanka [4]. The disease has been made notifiable since 2008 and a national action plan has been developed in 2008 to control leishmaniasis [16]. According to recent research findings and records of the Ministry of Health, CL is now recognized as an endemic disease in the country.

Leishmaniasis has been reported mostly in low altitude areas of Sri Lanka. Although large number of published studies is available on the burden, risk factors and molecular characteristics of vectors and parasites, investigations on seasonality and spatial distribution of leishmaniasis are not adequately documented on Sri Lanka. The knowledge of geographical spread of leishmaniasis in Sri Lanka is still limited. Therefore, the present evaluation was conducted to identify the seasonal variation and spatial distribution of leishmaniasis in Sri Lanka using data available from 2009 to 2016. This analysis could be used to validate and assist the reconsideration of control strategies and national or regional policies for Sri Lanka and for other countries where leishmaniasis is endemic. In addition, this could provide information on the climatic factors that may potentially favor its spread and identify risk areas through comparison with data presented by other researchers in different geographical areas and used in future epidemiological studies, including those on climatic change. This study also provides information how climatic changes in tropical countries effect for the epidemiology of neglected diseases.

## Methods

### Geography, population and climate

**Fig. 1:**
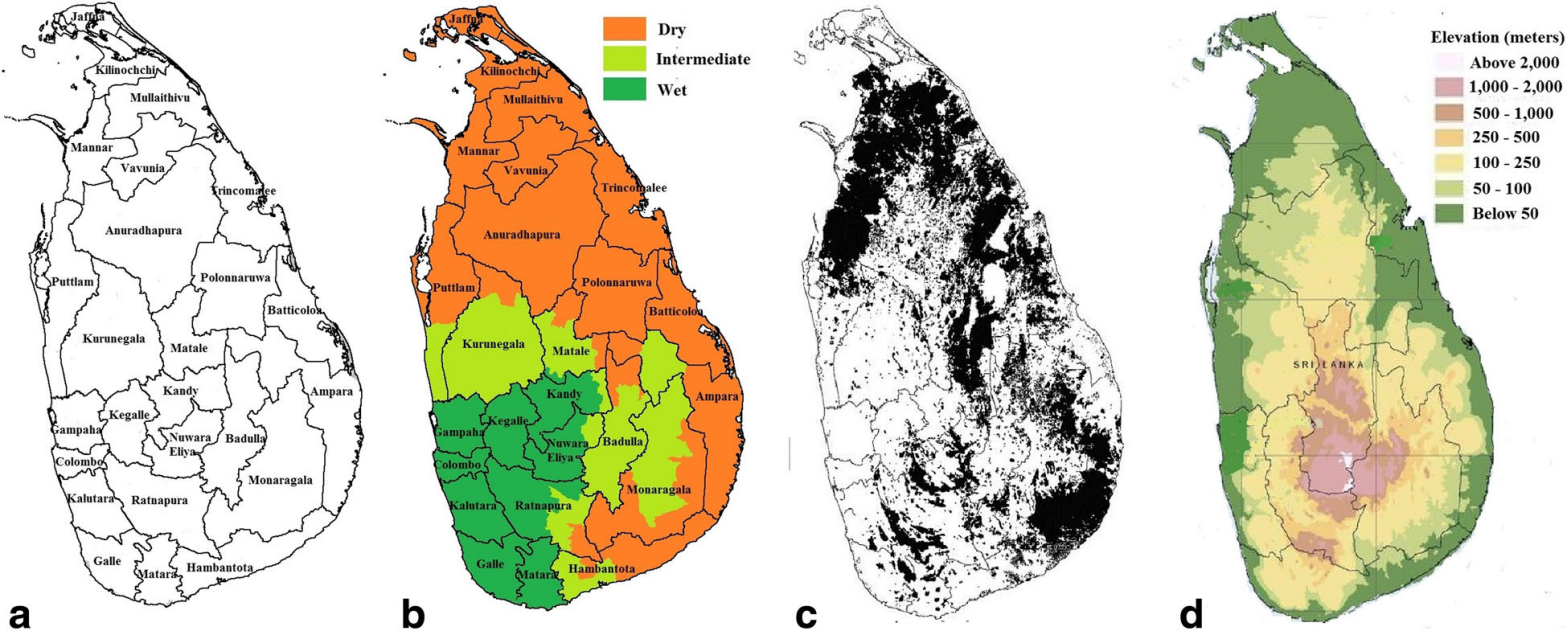
General characteristics of Sri Lanka **a** Administrative districts. **b** Major climatic zones. **c** Forest coverage (black shows forests in Sri Lanka). **d** Geography

Sri Lanka is a lower middle income country situated in the Indian Ocean with a long sea coast line between 5°55′ and 9°51′N, 79°42′ and 81°53′E, to the Southeast of India. The total population of Sri Lanka is about 20.2 million living in an area covering 65,610 km^2^ in 2011. Sri Lanka is divided into 9 provinces and 25 administrative districts (Fig. 1a). In Sri Lanka, 25% of the population is under 14 years and 85% of population resides in rural areas [17]. The central part of the island is mountainous with mountains ranging in altitude between 500 and 2500 m and the remainder of the island consists of lowlands. The mean annual temperature of low altitudes (0–100 m above sea level) in Sri Lanka varies between 26.5–28.5 °C and the temperature decreases with increasing altitude. The mean annual temperature of Nuwara-Eliya district situated at the highest altitudes (1500–2500 m) is 15.9 °C. Geographically, there are three climatic zones in Sri Lanka: western and southwestern areas form the wet zone; north-western and western slopes of the central mountain form the intermediate zone; and north, north-east, east and south-east areas form the dry zone (Fig. 1b).

In Sri Lanka, it was estimated that there was 26.6% of forest coverage in 2010 (Fig. 1c) [18]. Sri Lanka mainly consists of flat lands less than 300 m in elevation. However, rough plateau with hills and a range of moutains is in the south-central portion of Sri Lanka (Fig. 1d). Sri Lanka has a tropical climate and is affected by two major monsoons: From December to February, the north-east monsoon brings rain to the north, north-east regions and the eastern slopes of the central hill country and from June to October, the south-west monsoon brings rain to the south-west regions and central highlands [19]. April and August are the hottest months while January is the coolest month.

### Sampling of data

#### Leishmaniasis incidence data

Morbidity related data were obtained from a published series of weekly reports, quarter year reports and annual health reports published by the Epidemiology Unit and Health Statistics Unit of the Ministry of Health, Sri Lanka for the period of 2009 to 2016. Leishmaniasis was made a notifiable disease in Sri Lanka in 2008, and therefore, reporting of all persons with leishmaniasis to the relevant Medical Officer of Health (MOH) is a legal requirement.

#### Climatic and population data

Data (monthly) on temperature, rainfall, humidity, wind speed, cloud, sunny days and sun hours in Sri Lanka during the study period (2009–2016) were collected from the Department of Meteorology, Sri Lanka. The populations in administrative districts were ascertained from the Department of Census and Statistics, Sri Lanka for the study period.

### Data analysis

Reported cases of leishmaniasis were gathered from established worksheets and calculated cases for annual level and quarters of the year in the district level. Total human populations in the administrative districts were taken to calculate the leishmaniasis incidence per 100,000 people for 8 years from 2009 to 2016. Twelve months have been divided into four quarters as Quarter 1 (January, February, March), Quarter 2 (April, May, June), Quarter 3 (July, August, September) and Quarter 4 (October, November, December) to describe the seasonal fluctuations of disease incidences. Time series informations including number of leishmaniasis patients, trend and seasonal variations and seasonal adjusted leishmaniasis patients were analysed in each endemic districts from 2009 to 2016.

Data were entered into a Microsoft Excel data sheet and was analyzed by SPSS version 20 for statistical analysis. Descriptive statistics were calculated including percentage, mean and standard deviation to describe the univariate analysis. Pearson’s correlation coefficient analysis was applied to determine the association between climatic parameters and leishmaniasis incidence in endemic districts. Multiple regression method was used to determine the predictive effect of the climatic factors on the incidence of leishmaniasis. A 95% confidence level was used and *P*-value less than 0.05 was considered statistically significant.

Auto Regressive Integrated Moving Average (ARIMA) model was used to model and predict the incidence of leishmaniasis. Autocorrelation functions were used to to identify autoregressive functions. In time series analysis, the extent of the lag in an autoregressive model was identified by the partial autocorrelation function (PACF) for seasonal data.

## Results

### Spatial distribution of leishmaniasis

**Fig. 2:**
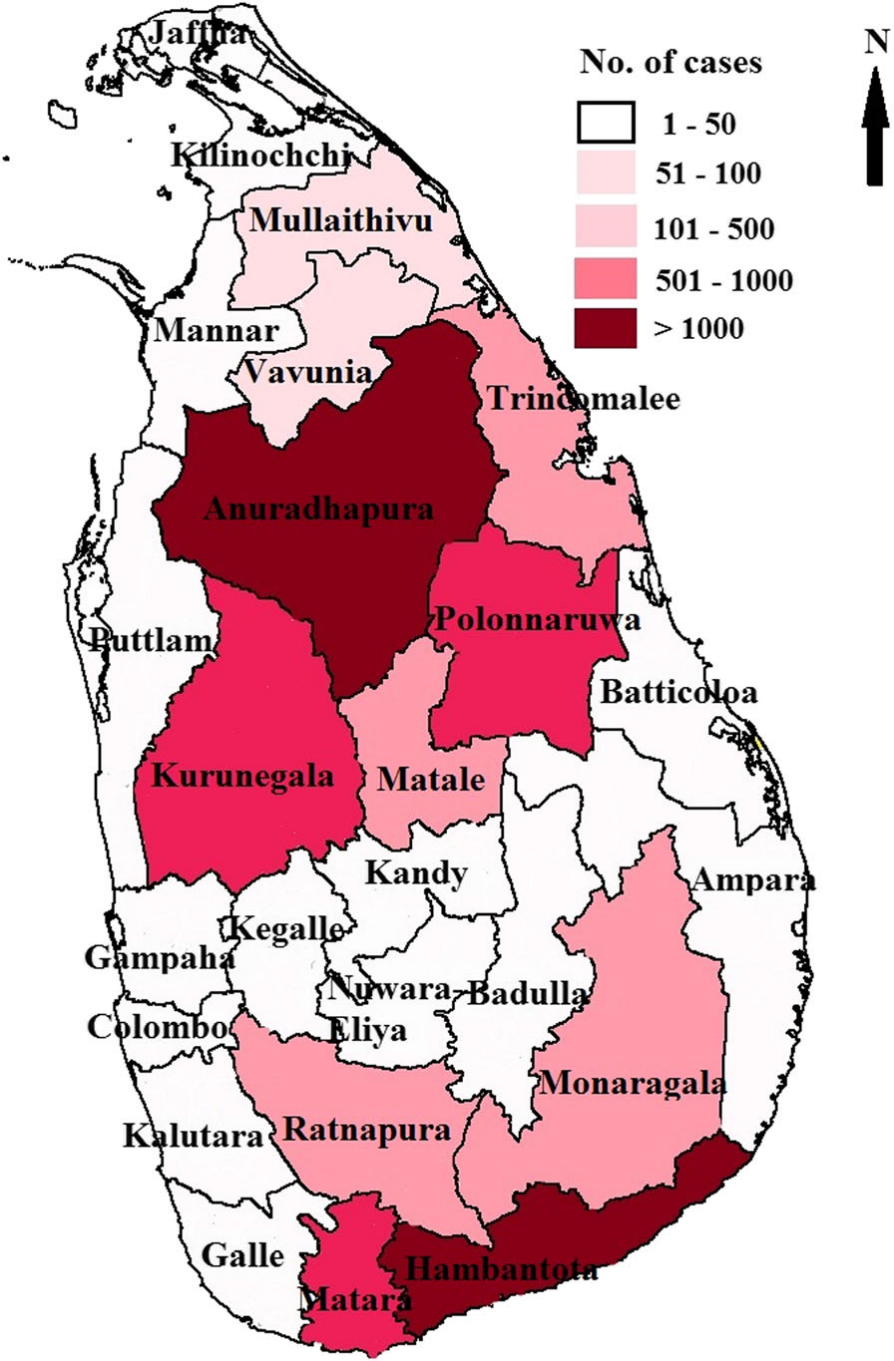
Number of leishmaniasis patients in all districts in the study period (2009–2016)

The total number of diagnosed leishmaniasis patients reported from 2009 to 2016 was 8487. Since 2012, more than 1000 cases have been newly identified annually and the highest number recorded in 2014 (Table 1). At the beginning of the study period, the disease was mainly concentrated in a few districts (Anuradhapura, Hambantota, Polonnaruwa and Matara) and with time, it spread to other districts (Fig. 2). Although leishmaniasis have been reported from all districts, Anuradhapura, Hambantota, Polonnaruwa, Kurunegala and Matara were the districts identified with more than 100 new patients annually. During the period of 2009–2016, the highest number of leishmaniasis patients was reported in the Anuradhapura district (2671) and it was 31.5% of all identified patients in this period. The second highest number of patients were identified in the Hambantota district (2465; 29%) followed by Polonnaruwa district (956; 11.3%), Matara district (833; 9.8%) and Kurunegala district (609; 7.2%). These five districts contributed around 90% of patients with leishmaniasis (7534, 88.8%) (Fig. 2). Out of these districts from 2009 to 2016, an 18.5-fold increase in *Leishmania* patients was shown in Kurunegala district followed by a 4-fold increase in the Polonnaruwa, and a 2.1-fold increase in Matara district. In addition, the number of leishmaniasis patients increased 10-fold in the Monaragala district (2010: 4 patients, 2016: 40 patients).

**Table 1:** Distribution of leishmaniasis cases in each district from 2009 to 2016

District	2009	2010	2011	2012	2013	2014	2015	2016
Colombo	2	3	1	4	1	3	1	0
Gampaha	2	3	0	1	5	3	3	7
Kalutara	1	1	1	3	0	0	0	0
Kandy	0	0	2	0	6	5	17	13
Matale	14	3	16	46	20	32	36	26
Nuwara Eliya	2	1	0	0	0	0	2	0
Galle	1	0	1	1	2	3	3	3
Hambantota	262	139	268	335	371	375	325	390
Matara	95	25	57	92	107	94	166	197
Jaffna	0	0	0	0	0	1	0	1
Kilinochchi	0	0	0	4	13	11	0	0
Mannar	0	0	0	4	4	5	1	0
Vavuniya	1	1	0	10	17	6	9	8
Mullaitivu	1	0	0	9	19	7	9	6
Batticaloa	0	0	0	2	0	0	0	1
Ampara	2	3	3	2	4	12	3	9
Trincomalee	12	11	9	17	30	9	6	18
Kurunegala	6	25	46	56	63	152	150	111
Puttalam	1	1	2	3	13	9	3	4
Anuradhapura	226	169	358	434	443	417	347	276
Polonnaruwa	34	26	156	143	178	153	130	136
Badulla	3	0	0	0	9	1	8	4
Monaragala	0	4	2	14	15	33	40	40
Ratnapura	11	8	18	35	18	34	19	1
Kegalle	0	3	0	2	2	2	0	3
Total	674	428	940	1219	1342	1367	1277	1240

Sources: Epidemiological data, Ministry of Health, Sri Lanka

Furthermore, the incidence of leishmaniasis patients per 100,000 people in Anuradhapura, Polonnaruwa and Hambantota districts rapidly increased during the period of 2010 to 2012. In addition, a rapid increase of patients was observed in Matara district from 2014 to 2016 (Table 1) and further to this, the notified cases of identified *Leishmania* patients gradually increased in Matale, Trincomalee and Mullaithivu districts. However, very low numbers of patients were identified in the western province (Colombo, Gampaha and Kalutara districts), Central highlands (Kandy, Nuwara-Eliya, Badulla and Kegalle districts) and the Jaffna Peninsula (Table 1).

**Fig. 3:**
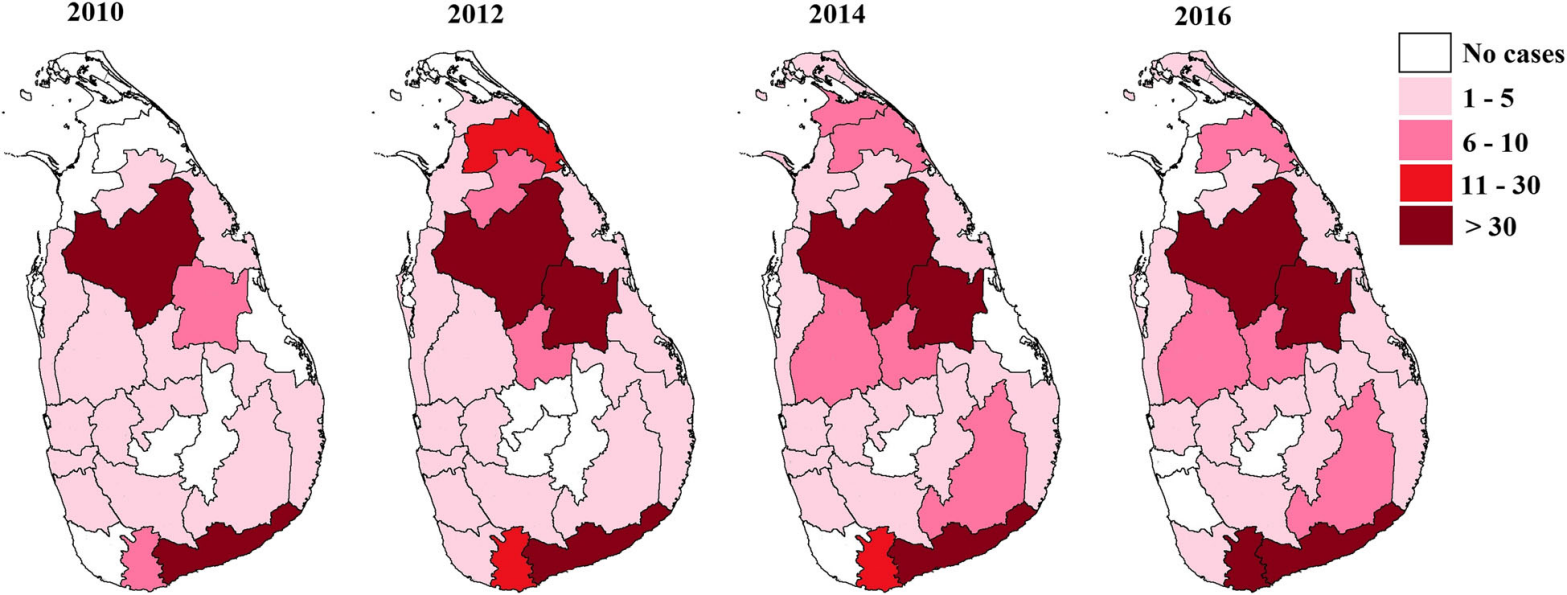
Incidence rates of leishmaniasis per 100,000 people (2010–2016)

When considering the incidence rate of leishmaniasis, more than 30 patients per 100,000 people were identified in Hambantota district in 2010. However in 2016, Hambantota (45.3), Anuradhapura (30.7), Polonnaruwa (32) and Matara (30.3) districts showed an incident rate of more than 30. In addition, the rate of leishmaniasis incidence in Kurunegala, Matale, Monaragala and Mullaithivu districts has become more than 5 per 100,000 people (Fig. 3).

### Seasonality and distribution of CL

**Fig. 4:**
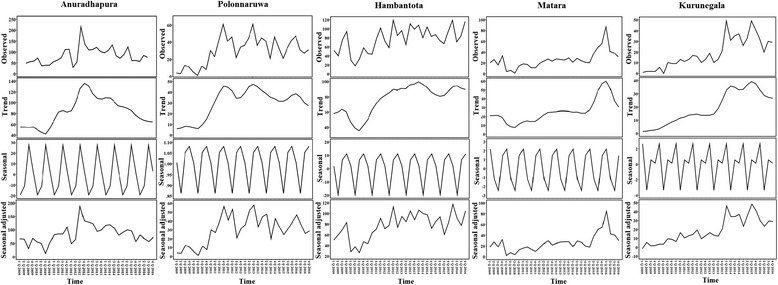
Decomposition of leishmaniasis time series into additive components in endemic districts from 2009 to 2016. From the top to the bottom: observed leishmaniasis patients, trend components, seasonal components and seasonal adjusted leishmaniasis patients

The top panel of Fig. 4 shows the distribution of the quartery cases of the disease cases of leishmaniasis in Sri Lanka between 2009 and 2016. The second panel is the trend component, which indicates a steady increase in leishmaniasis cases in Anuradhapura, Polonnaruwa and Hambantota districts from 2010 to 2012. Meanwhile, a sharp increasement of leishmaniasis cases in Matara and Kurunegala districts showed from 2014 to 2016. However, a decreasing trend of leishmaniasis cases showed in Anuradhapura district after 2012. The leishmaniasis incidence in Sri Lanka shows a seasonal trend, a peak in July to September, between the two monsoon periods. Meanwhile, during March-June there was a drop in the number of reported cased compared to the rest of the months. The third panel is the seasonal component, which shows the highest incidence rate in Anuradhapura district was reported during the third quarter (July-September). However, highest number of leishmaniasis patients in Polonnaruwa and Hambantota districts was reported in the fourth quarter (October-December). In addition, a similar number of new cases was identified in Matara district throughout the year since it is situated in wet zone. Meanwhile decreasing trends of leishmaniasis showed in north-central regions (Anuradhapura and Polonnaruwa districts) and increasing trends showed in north-western and southern regions (Hambantota, Matara and Kurunegala districts, Fig. 4).

### Correlation between climatic parameters and leishmaniasis cases

Correlation coefficients between the incidence of leishmaniasis and climate parameters were analyzed. Significantly positive correlations with monthly average and maximum temperatures were observed with the incidence of leishmaniasis in Hambantota and Matara districts. In addition, a significant negative correlation between leishmaniasis cases and humidity was observed in Matara district. Furthermore, wind speed and wind gust is positively correlated in Anuradhapura district. There was no correlation between leishmaniasis case incidence and any of the analyzed climatic parameters in Polonnaruwa and Kurunegala districts. In addition, negative correlations were observed between monthly average rainfall and leishmaniasis in Anuradhapura, Polonnaruwa, Hambantota and Matara districts (Table 2).

**Table 2:** Correlation between lesihmaniasis and monthly average climatic parameters in endemic districts

Climatic parameters	Anuradhapura		Polonnaruwa		Hambantota		Matara		Kurunegala	
	*r*	*P*-value	*r*	*P*-value	*r*	*P*-value	*r*	*P*-value	*r*	*P*-value
Minimum temperature	0.326	0.090	-0.114	0.564	0.214	0.274	0.251	0.197	-0.307	0.112
Maximum temperature	0.025	0.898	-0.074	0.714	0.494	0.008	0.591	0.001	0.357	0.062
Average temperature	-0.174	0.174	-0.063	0.751	0.417	0.027	0.684	< 0.001	0.173	0.378
Rainy days	-0.315	0.103	-0.082	0.678	-0.109	0.579	-0.121	0.540	0.113	0.566
Rainfall	-0.272	0.161	-0.163	0.407	-0.042	0.833	-0.418	0.027	0.106	0.591
Average wind speed (mph)	0.516	0.005	0.036	0.854	0.186	0.344	0.307	0.112	0.133	0.501
Maximum wind speed (mph)	0.596	0.002	0.061	0.607	0.359	0.060	0.134	0.498	0.185	0.345
Average wind gust (mph)	0.569	0.002	0.102	0.757	0.197	0.314	0.088	0.655	0.114	0.564
Humidity	-0.358	0.061	0.012	0.607	-0.033	0.869	-0.584	0.001	0.008	0.966
Cloud (%)	0.041	0.837	0.073	0.713	-0.253	0.194	-0.055	0.780	0.070	0.723
Sunny days	0.176	0.371	0.095	0.631	0.212	0.278	0.138	0.485	0.048	0.807
Sun hours	0.191	0.331	-0.143	0.469	-0.297	0.330	-0.086	0.660	-0.165	0.403

*Abbreviation*: *r* correlation coefficient

### Modeling and prediction of leishmaniasis incidence and climatic factors

Multiple regression analysis and autoregression analysis were used to identify a model and predictors associated with the incidence of leishmaniasis in endemic districts. Average wind speed was identified as a predictor in Polonnaruwa district. Meanwhile, humidity and maximum temperature were associated in Hambantota district. In addition, sun-hours were significantly associated in both ambantota and Kurunegala districts. However, no climatic factors were significantly found to be correlated with the incidence of leismaniasis in Anuradhapura and Matara districts (Table 3).

**Table 3:** Multiple regression method to model leishmaniasis cases using climatic variables

	Anuradhapura		Polonnaruwa		Hambantota		Matara		Kurunegala	
Climatic variables	Coefficient of variable	*P*-value	Coefficient of variable	*P*-value	Coefficient of variable	*P*-value	Coefficient of variable	*P*-value	Coefficient of variable	*P*-value
Constant	509.05	0.146	-478.52	0.493	-327.84	0.001	-567.43	0.960	-485.88	0.055
R-square	0.587		0.444		0.833		0.620		0.658	
Minimum temperature	-9.79	0.545	8.92	0.326	-0.02	0.742	2.95	0.760	0.01	0.903
Maximum temperature	25.37	0.376	-2.90	0.686	20.70	0.036	7.92	0.480	1.41	0.793
Average temperature	-21.95	0.302	3.76	0.676	-11.82	0.167	0.35	0.960	6.95	0.071
Rainy days	-3.78	0.533	1.12	0.699	5.58	0.084	6.76	0.110	0.67	0.640
Rainfall	-0.04	0.889	-0.13	0.313	-0.11	0.460	-0.15	0.180	0.00	0.911
Average wind speed	-4.53	0.543	-38.87	0.044	-4.26	0.794	2.70	0.260	3.44	0.565
Maximum wind speed	28.97	0.159	22.43	0.054	7.81	0.040	-3.17	0.780	-1.67	0.799
Average wind gust	-14.94	0.454	11.29	0.285	8.42	0.467	5.46	0.570	4.19	0.289
Humidity	-2.03	0.775	3.09	0.340	-3.04	0.019	2.08	0.610	-1.43	0.087
Cloud	-0.32	0.840	-0.45	0.701	2.33	0.593	-2.00	0.380	4.09	0.108
Sunny days	-6.76	0.288	0.04	0.989	0.70	0.802	4.96	0.270	0.96	0.467
Sun hours	-0.49	0.507	-0.30	0.582	-1.67	0.003	-0.18	0.770	-0.75	0.017

**Fig. 5:**
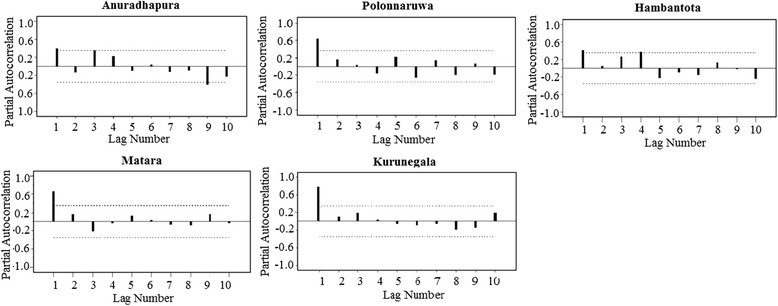
Autocorrelation plot of the “lag” (time span between observations) and the autocorrelation. The dotted lines indicated 95% bounds for statistical significance

An ARIMA model was used to analyze autoregression of seasonal data. The autocorrelation plots show a significant autocorrelation at lag 1 in all endemic districts. Further, partial autocorrelation function (PACF) shows from seasonal data insignificant spikes at the mentioned lags in Anuradhapura (9th lag) and Hambantota (4th lag) concluding no evidence of non-randomness process (Fig. 5). In addition, Polonnaruwa, Matara and Kurunegala districts had only one large significant spike at lag 1, which implies that we can discard the longer lags for those districts (Fig. 5).

## Discussion

Morbidity data are important for planning and evaluation of the health care system in a country. Sri Lanka is endemic for leishmaniasis caused by *Leishmania donovani*. For several decades, the public health awareness of the leishmaniasis and its impact on health was underestimated. To our knowledge, this is the first study in Sri Lanka to investigate the spatial distribution of leishmaniasis after it has been declared as a notifiable disease from 2008. At the beginning, the disease was mainly concentrated in a few districts, namely Anuradhapura, Polonnaruwa and Hambantota. Over the last decade, it spread out to other districts and number of recorded cases has sharply increased in Sri Lanka.

Although leishmaniasis has been reported from all districts, Anuradhapura, Hambantota, Polonnaruwa, Kurunegala, and Matara were the districts identified with more than 100 new patients annually. Out of them Anuradhapura, Polonnaruwa and Hambatota are situated in the dry zone while Kurunegala and Matara districts are located in the intermediate and wet zones, respectively. These results showed that there is an emerging epidemic of leishmaniasis in the southern, north-western and north-central regions of the island. The highest incidence was recorded in the Anuradhapura and Hambantota districts indicating an endemic hot spot affecting the provinces of north-central and southern Sri Lanka as its epicenter. These areas may be serving as source for leishmaniasis transmission from which the disease can spread to adjoining areas as well. Previous studies reported that the risk factors for leishmaniasis in endemic areas in Sri Lanka associated with the life style of the young male adults aged between 21 and 40 years having outdoor activities during the daytime [7, 16, 20]. People in this age group are most active due to their high level of social activity, occupation and education, thus the risk of exposure to sand fly bites is high. The distribution of leishmaniasis cases throughout the dry zone is unlike that of a recently introduced disease.

In addition to main endemic districts, the number of leishmaniasis cases reported in Matale, Ratnapura, Trincomalee, Mullaithvu and Monaragala districts has seen a gradual increase from 2009 to 2016. All of these are neighboring districts and the climatic and socio demographic characteristics are similar to that of major endemic districts. Movements of leishmaniasis patients and rervoir hosts from high risk areas to non-endemic areas may be the main factor for this finding.

Sand flies are most active from dusk to dawn. However, they are less active during the hottest times of the day. The female sand fly lays eggs in the bark and buttress roots of old trees, in ruined buildings, in cracks in house walls, in animal shelters and in household rubbish termite mounds and animal burrows having rich humus and moisture [21, 22]. These microhabitats provide shelter and breeding sites for sand flies and protect them from climatic changes such as sunlight, rain and wind. Populations of sand flies are generally localized and therefore individuals do not disperse far from their breeding sites [23]. Therefore, their breeding and resting places exist very close proximity to human settlements [24].

Agriculture related activities are the main livelihood of the inhabitants of these endemic areas. People in the dry and the intermediate climatic zones in Sri Lanka are mainly paddy farmers and are involved with chena cultivation during day time. The agricultural activity facilitates favorable resting and breeding habitats for sand flies, thus increasing the risk of infection [25]. Large scale irrigation systems throughout the endemic districts enhance the moisture rich soil and provide vector breeding places in agriculture lands [26]. Therefore, the abundance of sand fly population and breeding places can be found in agricultural lands close to human settlements, increasing the risk of disease. Previous studies have reported that most of leishmaniasis patients work in open environments such as agricultural farms and fields wearing clothes to cover only lower part of the body. Therefore, the exposure of sand fly bites to upper part of the body was higher compare to lower parts [27]. In addition, persons working in the armed forces have been identified as a high risk group to leishmaniasis [28]. The reason for its spread has been associated with the movement of military personnel into former uninhabited areas in north and north-central provinces due to the civil war [29].

Studies conducted in the southern region (Hambantota and Matara) reported that household clustering and poor housing conditions were associated with leishmaniasis, favoring a peridomestic transmission [8, 30]. Poverty plays a major role in facilitating poor sanitary and housing conditions which contribute to enhancing peridomestic sand fly breeding and resting sites [31]. More than 80% of houses in the rural sector in Sri Lanka still use firewood for cooking while only one third of urban households use wood [17]. Therefore, a majority of rural women often go to forests and scrub jungles to collect firewood. They therefore have a risk of exposure to sand fly bites because of the abundance of sand fly breeding sites present in rural areas. Geographically, the majority of scrub jungles and forests present in north, north-central, north-western and southeastern areas are the regions where leishmaniasis is endemic. Frequent visits and activities related to scrub jungle have been identified as a risk factor for *Leishmania* infection [28]. Most leishmaniasis is zoonotic and humans become infected only when accidentally exposed to the transmitting sand flies.

Previous studies reported that the occurrence of leishmaniasis is seasonal [32–34]. Climatic changes in rainfall, humidity and atmospheric temperature influence behavioral activities and the life-cycle of vectors and reservoir hosts for leishmaniasis [35, 36]. A study in India reported that 29 °C to 31 °C was the most favorable range of the distribution of leishmaniasis, suggesting the potential of *P. argentipes* abundance in higher temperatures [37]. In the present study, we identified that all of the endemic regions are situated in low altitude areas less than 100 m above sea level and the mean temperature of all of these districts range between 26.5–28.5 °C. In Matara and Hambantota districts in the southern province, we observed a positive correlation between leishmaniasis and the mean and minimum average temperatures. In addition, the correlation between all other climatic parameters with the incidence of leishmaniasis was similar in Matara and Hambantota districts. This may be due to both of these districts being neighboring districts and having similar climatic characteristics. Similarly, a study in Bangladesh observed that a high incidence of the disease occurred during the warm months [22].

A previous study reported that *P. argentipes* is present at almost all altitudes in Sri Lanka [38]. However, this study shows that the highest incidence of leishmania patients was found less than 100 m above sea level. Another study identified the highest density of *P. argentipes* in the southern province where leishmaniasis is an emerging epidemic disease [39]. In addition, a recent study in Iran showed that optimal atmospheric conditions for breeding and maturation of sand fly vectors are present in low altitude areas [40]. In the present study, we identified that the incidence of leishmaniasis at high altitudes is very low compared to the cases identified from areas situated in low altitudes. Temperature gradually decreases with an increase in altitude. Sand fly prefer to live in environments with high humidity of 70–100% and rainfall helps to increase relative humidity [41, 42]. With increasing altitude, the air gets cooler resulting in low humidity at high altitudes. Therefore, the environmental conditions in mountainous regions are unfavorable for the breeding and maturation of sand flies. In addition, lesser human activity on the environment at high altitudes minimizes the exposure of sand fly bites at high altitudes [38]. Similarly, studies in India and Turkey reported that the abundance of sand flies showed a negative correlation with an increase in altitude [43, 44]. Another study in India identified that the number of kala-azar cases decreased with an increase in altitude and no kala-azar case was recorded beyond the 300 m in altitude, suggesting that altitudes play a major role in the distribution of the sand fly [24].

In the present study, a greater number of leishmaniasis cases were reported in dry seasons (July-September). This was consistent with studies in Colombia and Ethiopia, which reported that the increased frequency of droughts is likely to increase the incidence of the disease [45, 46]. In dry conditions like droughts, people in rural areas cluster around water supplies because of the limited water sources, exposing them to a high concentration of vector populations. Bhunia et al. [24] reported that there was a strong association between visceral leishmaniasis and water bodies and showed a decreasing trend of the disease with increased distance from water bodies in India. Other studies in India and Bangladesh reported that the incidence of leishmaniasis of flood controlled areas was significantly higher than flooding areas, suggesting that flooding removes organic matters and vector breeding sites [47, 48]. Interestingly, this fact is negatively correlated with increased rainfall in endemic regions. One of the possible reasons for this relation is that high humidity in dry seasons contributes breeding and dissemination of sand fly vectors [48]. In addition, increasing movements of humans in cultivated and forest areas during dry seasons might contribute to an increase in the exposure to sand fly bites.

Finally, we suggest that the transmission of leishmaniasis is linked by human behavior of outdoor activities under climatic conditions favoring the maturation and distribution of sand flies. Future studies of bionomics on vector, parasite and reservoir hosts, morphological variations of the infection will be important to understand the epidemiology of leishmaniasis in Sri Lanka. There were several limitations in this study. The present reporting and recording of data as a total count per district may not reflect the actual case burden. In some instances the persons with the disease were limited only to certain clusters. In addition, the true correlations cannot be calculated due to the following reasons: patient records represent as total for the whole district (No GNDs or MOH) and climatic data represent only one station. Significant variations in different micro-climate and micro-level habitats that may be present in individual districts could not be included in this analysis. Therefore, this analysis may misinterpret the actual epidemiology of the disease. We hope to discuss these issues with the Ministry of Health and provincial ministries of health to review and improve the data collection systems for this important public health problem.

## Conclusions

Leishmaniasis is endemic in north-central, north-western and southern regions of Sri Lanka. People living at low altitudes and close to paddy fields and forests have a high risk of acquiring the disease. Temperature, humidity and wind speed contribute to transmission of leishmaniasis in endemic regions. In addition, there is a negative correlation between rainfall and leishmaniasis in Matara district. Health education of the disease, both for public and health workers, identifying reservoir hosts and vector control will be important initiatives to prevent the spread of this disease in Sri Lanka.

## Abbreviations

CL: Cutaneous leishmaniasis
DHS: Demographic and health survey
VL: Viceral leishmaniasis
WHO: World Health Organization
